# Physiology of lived experience: Boston wrong: A series of physiological calamities at the Boston Marathon

**DOI:** 10.1113/EP093154

**Published:** 2025-10-27

**Authors:** Daniel H. Craighead

**Affiliations:** ^1^ School of Kinesiology University of Minnesota Minneapolis Minnesota USA

Even under ideal conditions and with excellent preparation, completing a marathon run (42.2 km) near the limit of one's abilities represents a significant challenge for human physiology. For example, during a marathon race, many runners experience gastrointestinal distress or fluid and electrolyte imbalances, and there is a high risk for musculoskeletal injury. After completing a marathon, biomarkers of skeletal muscle and myocardial damage, and acute kidney injury, are often elevated. Additionally, transient impairments in respiratory function, and altered immune function contributing to an increased risk for upper respiratory tract infections, are observed post‐race (Braschler et al., 2025). Thus, the marathon represents a substantial test of the body's ability to respond to stress.

The World Marathon Majors represent a collection of seven of the largest and most prestigious marathons in the world. The original World Marathon Majors are the Boston, London, Berlin and Chicago Marathons, with the Tokyo and Sydney Marathons added later. Each of the World Marathon Majors poses a unique set of challenges based on factors such as course elevation profiles and climate. For example, the Boston, New York and Sydney Marathon courses feature significant gains and losses in elevation relative to the other World Marathon Majors, which are generally flat and fast. This editorial will focus on my personal experiences at the Boston Marathon.

The Boston Marathon is one of the world's oldest marathons, with its first running in 1897 (Thompson & Venero, 2009). Run in mid‐April, the Boston Marathon features a point‐to‐point course that includes over 240 m of elevation gain and 380 m of elevation loss (Figure 1). These features lead to greater variability in performances at the Boston Marathon compared to other World Marathon Majors (Maffetone et al., 2017). This can partially be explained by the large variability in weather conditions experienced in Boston in springtime and the large impact of wind direction on race times on a point‐to‐point course (Knechtle et al., 2019; Maffetone et al., 2017). Hot temperatures impair performance by necessitating the redistribution of blood flow from the active skeletal muscle to the skin and increasing fluid loss through higher sweat rates, both responses needed to prevent a dangerous increase in body core temperature (Maughan, 2010). On the other hand, cold temperatures likely impair endurance performance by reducing muscle function (Castellani & Tipton, 2015), whereas headwinds increase the metabolic cost of running (Beaumont & Polidori, 2025).

**FIGURE 1:**
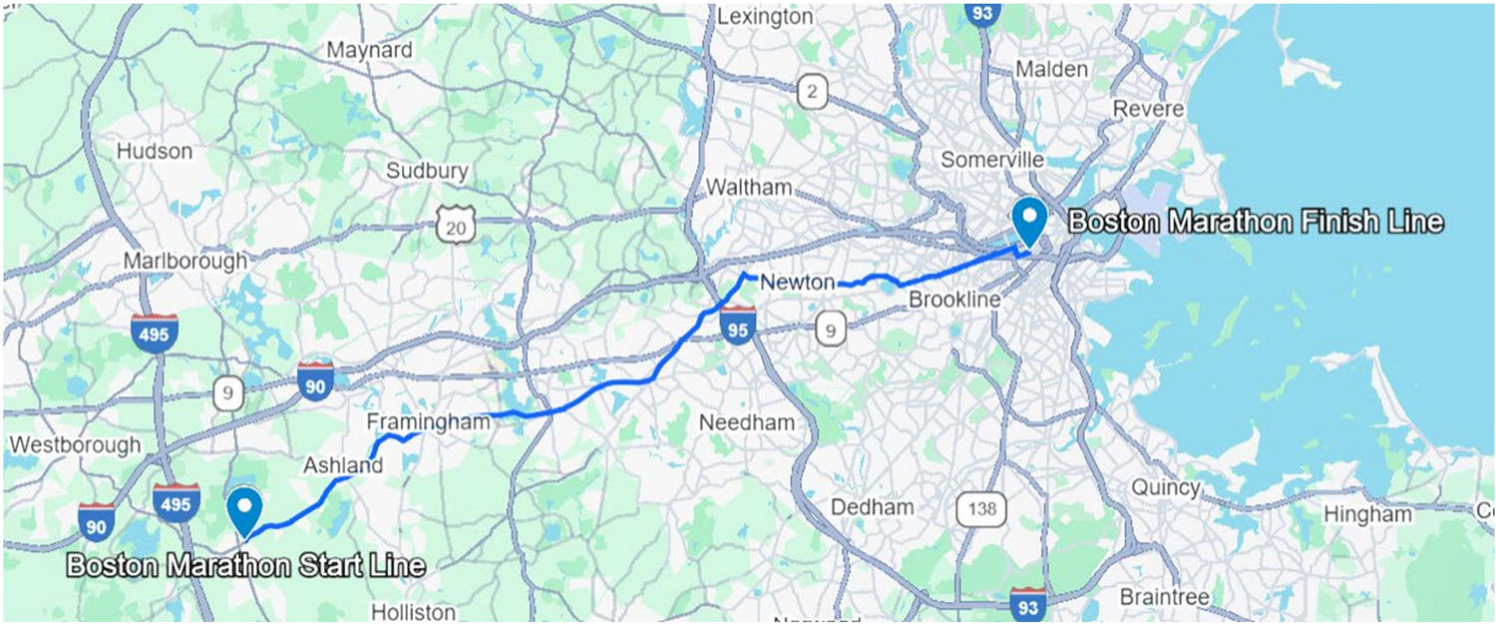
Course map of the Boston Marathon. The course runs west to east, starting in Hopkinton and ending in Boston Massachusetts. Map data © 2025 Google.

The weather and course profile often are not the only factors impacting physiological function, and hence athletic performance, on race day. As many marathoners experience firsthand, rarely is a marathon run with perfect preparation. For example, training time lost to injury is a common challenge for avid marathoners. I have had the opportunity to start the Boston Marathon on three occasions, and on each occasion faced a unique set of physiological stressors, in training and on race day, that greatly impacted my performance. Some of these challenges would be expected when training for and racing a marathon, while one is quite uncommon. The stories behind those three Boston Marathon experiences are as follows.

I began running marathons while in graduate school and the 2015 Boston Marathon was my second marathon. After a successful marathon debut in the fall of 2013, I increased the intensity of my training for Boston with hopes of a high finish and setting a personal best. Training was going well, but after running >160 km per week for multiple weeks in a row, my energy levels started to wane, and the quality of my runs began to suffer. Cutting back on training did not help and any attempts at high‐intensity running quickly resulted in my slowing to a trot. At this point it was 6 weeks until race day, so I needed a solution quickly.

A blood test revealed a mild case of iron deficiency anaemia, likely brought on by a combination of my high‐volume of training and pizza‐based diet. Iron is a critical component of haemoglobin, the oxygen‐carrying protein within red blood cells (Moustarah & Daley, 2025). Without adequate iron, oxygen carrying capacity is reduced. As a result, during endurance exercise, insufficient oxygen is delivered to the skeletal muscle, which limits aerobic energy production and impairs endurance exercise performance (Sim et al., 2019). In short, it was bad news for my goal of setting a personal best. I immediately began taking daily iron supplements to reverse my iron deficiency and could feel my energy returning as race day approached. While my training was limited for a few weeks, with timely restoration of my iron levels I was able to rebound. I was able to race competitively and even set a 26‐s personal best. Despite the substantial impact that iron deficiency played in my preparation, my first Boston Marathon was overall a success.

I returned to Boston in 2018 following my relocation to Boulder, Colorado for a postdoctoral fellowship in June of 2017. Located 1600 m above sea level, Boulder is the home of many of the world's best endurance athletes, who move there seeking the purported performance‐enhancing adaptations, such as increasing blood haemoglobin concentrations and muscle buffering capacity, that come with living and training at high altitude (Bailey & Davies, 1997). Thus, after spending nearly a year living and training in Boulder (and taking my iron supplements), I felt confident that I would achieve a new personal best in Boston.

However, with race day temperatures around 4°C, heavy rainfall, and headwinds gusting over 40 km/h, conditions were less than ideal for running fast. I could feel myself getting colder on the start line, as the conditions were more extreme than by body could counteract through cutaneous vasoconstriction and thermogenesis via shivering (Kenney & Munce, 2003). I had also failed to behaviourally thermoregulate properly, as I was standing on the start line in shorts and a rain‐soaked jacket. This was quite an embarrassing mistake. I had only just recently completed my PhD with Drs Larry Kenney and Lacy Alexander, experts in human thermoregulation, and should have been more prepared. My only hope was that the increase in metabolic heat production that would come once the race started would be enough to warm me up. Unfortunately, like so many runners that day, even after starting to run, my body core temperature continued to drop. Being a physiologist came in handy, as I was able to recognize cold in my extremities, the loss in muscle power and coordination, and the clouded cognition I was beginning to feel as markers suggesting my body was going into hypothermia (Castellani et al., 2021). I had over half of the race to go and I knew that trying to make it to the finish line would be dangerous. I also knew that stopping at an inopportune location, and lowering my metabolic heat production without being able to escape the cold weather, would be similarly problematic. I ran on, though with no memory of exactly how long, as the worsening hypothermia was more severely impacting my brain function. Thankfully, I saw a race medical tent at some point around the halfway mark. I veered into the medical tent and ended my race. Not surprisingly, I was far from the only person in the medical tent, which was quickly overrun with other chilled runners. A nearby church graciously opened their doors and served as a rewarming centre for the dozens of us that were abandoning the race until school buses could be procured to transport us all to downtown Boston.

I came back to Boston in 2019, ready to make up for my weather‐induced dropout in 2018. Training had gone very well, and the weather was much more agreeable than the year prior. Everything was in place to have a great race. However, this time the surprise factor that impacted by race was … impact! The start gun went off, but only a few metres after crossing the start line a runner a few rows in front of me tripped and fell. In the forward‐accelerating mass of shoulder‐to‐shoulder runners, there was no way to avoid what happened next. Like dominos, the runners in front of me toppled over, until it was my turn to trip over the people on the ground. Down I went but thanks to the adrenaline coursing through my blood stream, I popped right back up off the ground. As I did not feel any pain, I brushed off the rough start and continued my course thinking I was uninjured; however, this was just my body's stress‐induced analgesic response protecting me from pain following a severe injury (Butler & Finn, 2009). When I finally looked down, thanks to the concerned looks and words of the runners beside me, I found that the flat plane on which my hand and wrist once lived now looked more like a dinner fork.

I didn't know it yet, but I had sustained a displaced fracture of my radius and displaced an unknown number of carpals. Though I still did not feel any pain, I quickly ran off the course towards a first responder. My entire race lasted less than 30 s. I felt the oncoming symptoms of vasovagal syncope (blurry vision, hyperventilation, diaphoresis) (Fenton et al., 2000) as the EMTs arrived. In my mind, I ‘expertly’ articulated to the EMTs my forthcoming loss of consciousness and requested to move into a supine position. In reality, my subsequent fainting suggested I was experiencing cardiogenic shock and delivering inadequate oxygen to my brain (Vincent & De Backer, 2013), so my communication was likely less than clear. The next thing I remember, I woke up on the ground. I was quickly loaded into an ambulance and taken to the hospital. The next week, I would have a metal plate and over a dozen screws inserted into my wrist to realign the bones (Figure 2) – a rather severe contact injury from a, supposedly, non‐contact sport.

**FIGURE 2:**
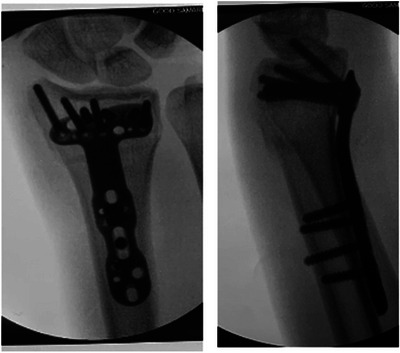
Metal plate and screws used to repair the author's displaced radial fracture obtained during the 2019 Boston Marathon.

I have not attempted the Boston Marathon since 2019. As correlation does not equal causation, the observed inverse association between year and the percentage of the race I completed *should not* predict future outcomes. However, I have decided to play it safe as I am not personally ready to risk even greater injury. Thankfully, this relation seems to apply only to the Boston Marathon, as I completed plenty of other marathons without incident in the following years. In conclusion, there are a host of physiological challenges inherent to completing a marathon to the best of one's abilities and these challenges can be compounded due to the unpredictable nature of health, weather and impact forces. To those running Boston in the future, I wish you more luck than I have had. But please, do not break a leg (or arm)!

## AUTHOR CONTRIBUTIONS

Sole author.

## CONFLICT OF INTEREST

None declared.

## FUNDING INFORMATION

None.
